# The adaptation of older adults’ transition to residential care facilities and cultural factors: a meta-synthesis

**DOI:** 10.1186/s12877-020-01987-w

**Published:** 2021-01-18

**Authors:** Changxian Sun, Yaping Ding, Yan Cui, Shuqin Zhu, Xianwen Li, Shen Chen, Rong Zhou, Yiting Yu

**Affiliations:** 1 Nanjing Medical University, 101 Longmian Avenue, Jiangning District, Nanjing, 211166 P.R. China; 2 Jiangsu Vocational Institute of Commerce, Nanjing, China

**Keywords:** Aged, Adaptation, Long-term care, Residential facilities, Transition

## Abstract

**Background:**

The transition to residential care facilities can be stressful for older people, entailing numerous challenges. Many qualitative studies focused on the adjustment and the experiences associated with older adults’ admission to residential care facilities. However, there have been few studies to synthesize qualitative studies and pay attention to the cultural factors influencing adaptation. The aim is to appraise the adaptation of older people’ s transition to the residential care facilities.

**Methods:**

We followed the method of Preferred Reporting Items of Systematic Review and Meta-Analysis (PRISMA). Six databases (CINHAL, Cochrane, Embase, Pubmed, PsycInfo, and Web of Science) were searched systematically from their inception until April 2020 using Medical Subject Headings (MSH) or Subject Headings plus free-text words. The CASP evaluation for qualitative studies was used for quality appraisal and meta-aggregation was used in the data analysis.

**Results:**

Ten studies (from 7 countries on 3 continents) were included in this review. We synthesized two main findings: the impacts of culture on adaptation and the transition process.

**Conclusions:**

Understanding the cultural factors helps nursing staff to gain new insight into older adults’ transition to residential care facilities. The consideration of cultural factors might be incorporated into tailored interventions for residents during transition. Nursing staff is advised to pay attention to the decision-making process before residents’ admission to the residential care facilities, and care plans are best made by residents, family members, and staff members together at the beginning of the decision-making process.

**Supplementary Information:**

The online version contains supplementary material available at 10.1186/s12877-020-01987-w.

## Background

The ageing of the population is a global challenge. Ageing causes both frailty and disability. More older adults require more extensive care than family members can provide. In recent years, self-care is declining because of exacerbated health, falls, the inability to keep up with household tasks, and lack of support [1]. Furthermore, the traditional family structure and socialization have changed, and more women work in the paid labour force because of urbanization and industrialization; for these reasons, home-base care for older adults is declining. The demand for residential care facilities (RCFs) has increased rapidly.

The transition to RCFs is a critical period for older people. This transition is a significant life event that requires older adults to adapt to a new environment. They must confront substantial challenges. Previous studies have reported that residents experienced substantial emotional responses, limited communication opportunities, isolation, and changes in social support and life patterns [2]. In particular, newly admitted residents also experienced the loss of autonomy, stress, and uncertainty at the beginning of the relocation [3]. Older people are more vulnerable to such novel places because they are usually highly dependent on those familiar to them and their habitual environment for maintaining their independence [4]. Maladjustment to their new situation harms on the quality of life and health status of the older adults [3].

Meleis defined transition as a passage or movement from one state, condition, or place to another, containing developmental transitions, situational transitions, and health-illness transitions. The transition of an older adult from home to a residential care facility as a situational transition [5]. Brooke described newly admitted older people’s transition process as follows: disorganization, reorganization, relationship building, and stabilization [6]. Wilson reported that the process included the overwhelmed phase, the adjustment phase, and the initial acceptance phase [7]. According to Meleis, the transition to RCFs involves a change of location from home or elsewhere to the RCFs, a process that includes not only postadmission but also preadmission. The nursing home life is continuous with residents’ past life, and the preparation of the preadmission process leads to either postadmission acceptance or resistance [8]. Iwasiw also described transition activities as relevant to deciding where to live, moving, trying to make the nursing home like home, maintaining previous relationships, beginning new ones, and fitting in [9]. However, Brooke and Wilson did not incorporate the period of preadmission into the transition process. Therefore, a recommendation to extend or refine the transition process is made in this study.

Culture is seen as an important factor influencing how older adults respond to nursing home life [10]. The RCF is a place of residence for older adults from different socioeconomic statuses, educational levels, careers, and cultural backgrounds [11]. Older adults in different countries and from different cultures respond differently to relocation. One’s lived experience is influenced by cultural values and the social context [10]. In recent years, some nursing homes have become more sensitive to residents’ cultural needs; however, most caregivers are so busy providing basic care that they can easily neglect the cultural aspects of residents’ lives [12].

Another study offered a meta-synthesis focusing on the experience of older adults’ transition to RCFs, specifically covering settings in the USA and Canada [13]. This study aimed to synthesize qualitative research studies to supplement the connotation of the transition process, and help nursing staff to better understand older adults’ transition to RCFs and the cultural factors influencing adaptation.

## Methods

### Eligibility criteria

The studies were included in the review if they met the following criteria: (1) employed qualitative collection and analysis methods; (2) were focused on the adaptation to the RCFs; and (3) included older adults who could speak and understand clearly in the sample. The RCFs included nursing homes, nursing care facilities, assisted living facilities, residential care homes, care homes, and long-term care facilities in this study.

### Search strategy

A systematic literature search was conducted in CINHAL, Cochrane, Embase, Pubmed, PsycInfo, and Web of Science from their inception to April 2020 using Medical Subject Headings (MSH) or Subject Headings plus free-text words. The thesaurus vocabulary of each database was utilized to adapt the search terms (Table 1). Those articles that met the inclusion/exclusion criteria and were eligible for quality appraisal also had their reference lists searched. The initial search retrieved 493 references. Five additional records were identified from other resources as supplements.

**Table 1:** Search terms. Terms used for search strategy across all databases using elements from the review question. PICo (Population, Phenomenon of Interest, Context)

Population	Context	Phenomenon of Interest	Study Design
“Aged [Mesh]” OR“older adults” OR “older persons” OR “elderly individuals” OR “elderly people”OR “elderly residents” OR residents	(relocation OR transition OR admission)AND (“Residential Facilities”[Mesh]) OR “Homes for the Aged”[Mesh]) OR “Long-Term Care”[Mesh]) OR “aged care home” OR “long-term care facility” OR “LTC facilities” OR “residential aged care facility”)	“Social Adjustment”[Mesh] OR (adaptation OR adjustment OR “psychosocial changes” OR “psychosocial adjustment” OR adapt* OR adjust*	“nursing methodology” OR “case study” OR “constant comparison” OR “content analysis” OR “descriptive study” OR “discourse analysis” OR ethnography OR exploratory OR feminist OR “focus group” OR “grounded theory” OR hermeneutic OR interview OR narrative OR naturalistic OR “participant observation” OR phenomenology OR “qualitative method” OR “qualitative research” OR “qualitative study” OR “thematic analysis”

The above table used Pubmed as an example

### Selection of articles

**Fig. 1:**
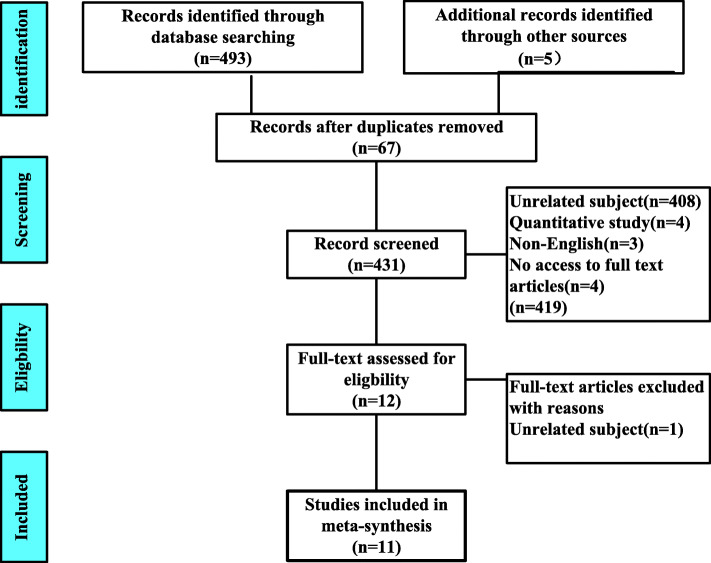
PRSM Results

The ‘Preferred Reporting Items of Systematic Review and Meta-Analysis’ (PRISMA) [14] was used to filter the filtering process of the records (*N* = 498). Following the removal of duplicates, the titles and abstracts of all remaining papers were screened blindly by two reviewers. Conflicts were resolved by discussion among the authorship team to achieve a consensus. Fourteen papers were chosen for full-text review. After the full texts were reviewed by the two authors, one articles were removed (Fig. 1).

### Quality appraisal of studies

The quality of papers was assessed using the Critical Appraisal Skills Programme [15] assessment tool for qualitative research, which consisted of 10 questions. Each question was given a score based on a response of ‘ye’, ‘no’, or ‘cannot tell’; every yes response deserved a score of 1; summation scores < 6 were not included in the analysis [13]. Two authors appraised each study independently, and disagreements were resolved through consultation with the team of authors. The consensus was achieved for all the studies. One paper was excluded because its score was 5 [16]. (Table 2).

**Table 2:** Critical appraisal tool for qualitative studies

Author(s)/year	Clear research aims	Qualitative methodology appropriate	Research design appropriate	Recruitment strategy appropriate	Data collection appropriate?	Participant Researcher relationship considered	Ethical issues considered?	Data analysis sufficiently rigorous	Clear statement of findings?	How valuable is the research?	CASP score
Tracy [17]	Yes	Yes	Yes	Yes	Yes	Yes	Yes	Yes	Yes	Yes	10
Wilson [7]	Yes	Yes	Yes	Yes	Yes	Can not tell	Yes	Yes	Yes	Yes	9
Heliker [18]	Yes	Yes	Yes	Yes	Yes	Yes	Yes	Yes	Yes	Yes	10
Koppitz [19]	Yes	Yes	Yes	Yes	Yes	Can not tell	Yes	Yes	Yes	Yes	9
Lee [10]	Yes	Yes	Yes	Yes	Yes	Can not tell	Yes	Yes	Yes	Yes	9
Guevara [20]	Yes	Yes	Yes	Yes	Yes	Can not tell	Yes	Yes	Yes	Yes	9
Iwasiw [9]	Yes	Yes	Yes	Yes	Yes	Can not tell	Yes	No	Yes	Yes	9
Lee [21]	Yes	Yes	Yes	Yes	Yes	Yes	Yes	Yes	Yes	Yes	10
Kahn [22]	Yes	Yes	Yes	Yes	Yes	No	No	Can not tell	Yes	Yes	7
Križaj [23]	Yes	Yes	Yes	Yes	Yes	Can not tell	Yes	Yes	Yes	Yes	9
Salarvand [16]	Yes	Yes	Yes	Yes	Can not tell	Can not tell	No	No	No	Can not tell	5

### Data extraction

Ten studies [7, 9, 10, 17–23] were included in the final synthesis. Five studies were undertaken in North America (4 from the US and 1 from Canada), three in Europe (1 from the UK, 1 from Switzerland, and 1 from the Republic of Slovenia), and two in Asia (1 from Hongkong, China; and1 from the Philippines).

One author independently extracted study characteristics and demographics using an author-devised data extraction sheet, including first author surname, year, country, sample, research purpose, methodology and findings (Table 3).

**Table 3:** Summary of the studies included in the review

Authors	Country	Sampling	Research Purpose	Method	Collection	analysis	Classification of the Findings
Tracy [17]	USA	No.28, Gender:9 M/19F Age:68–93,	To explore feelings about their transitional experience and recall situations in their adjustment process	Hermeneutic phenomenology	Group interview	Van Manen’s method	1Self-motivated move versus familial encouragement; 2Ties to the past versus starting a new; Independence versus dependence; 3Affection versus disdain; 4Adjustment versus maladjustment;
Wilson [7]	USA	No.15, Gender:4 M/11F Age:76–97	To identify variance in the initial responses of older adults whose move to a nursing home is either planned or unplanned	Grounded theory	In-depth semi-structure Interviews, observation	Constant comparative method	1Overwhelmed phase 2Adjustment 3 Initial accept phase
Heliker [18]	USA	No.10 Gender:Not describe Age: Not describe	To describe the phenomenon of being admitted and living in a nursing home;to explicating their experience of transition	Hermeneutic phenomenology	Interviews observation	Heideggerian	1Becoming homeless; 2Getting settled and learning the ropes; 3Creating a place;
Koppitz [19]	Switzerland	No.31 Gender:8 M/23F Age:83.1 ±6.2	To examine the process of an uplanned admission into a nursing home and its impact on the individuals’ adaptation	Descriptive phenomenology	semi-structure Interviews	Mayring’s approach of content analysis	1Being cared for; 2Moving on; 3Being cut off; 4Being restricted;
Lee [10]	Hongkong China	No.18 Gender:9 M/9F Age:70–96	To describe the process through which residents in Hongkong adjust nursing home placement	Grounded theory	In-depth Interviews	Constant comparative method	1Orienting 2Normalizing 3Rationalizing 4Stabilizing
Guevara [20]	Philippines	No.20 Gender:7 M/13F Age:69–84	To examine the process of acclimatization of Filipino elderly in a nursing care facility	Grounded theory	In-depth unstructured Interviews	Constant comparative method	1Reminiscing phase 2Recommencing phase 3Reinforcing phase 4Recapturing phase 5 Renewing 6Rekindling Phase
Iwasiw [9]	Canada	No.12 Gender:2 M/10F Age:67–96	To investigate the experience of this early phase of relocation	Not describe	interviews	Constant comparative method	1Emotional reactions 2Transition activities 3Reflecting on their situation 4Connecting with a personal philosophy
Lee [21]	UK	No.8 Gender:Not describe Age:65–97	To explore old pepole’s experience of transition into residential care	Exploratory design	interviews	Narrative	1Reflecting key plots of ‘control’, ‘power’, ‘identity’ and ‘uncertainty’ interwoven throughout their narratives; 2Experiencing some difficulties in incorporating this transition into their life stories.; 3Not feeling confident in their decision to move; 4Living in constant fear of losing their memory; 5Limited expectations for their future
Kahn [22]	USA	No.21 Gender:2 M/19F Age:66–93	To describe the process successfully used to adapt to nursing home environment	Ethographic study	interviews observations	Narrative	1Recognizing the ambivalence 2Downplaying the negative 3No other Options 4An act of will
Križaj [23]	Republic of Slovenia	No.6 Gender:3 M/3F Age:74–92	To explore Slovenian older people’s experiences of transition into a care home	Phenomenological approach	Semi-structured, in-depth interviews	Interpretative Phenomenological Analysis	1This is who I am, 2Adjusting my daily occupations, 3The value of health

### Data analysis

Meta-aggregation was used in this study. It involves identifying findings, grouping findings into categories, and grouping categories into synthesized findings [24]. The researcher read, repeatedly analyzed the findings of each study, summarized and combined similar findings to form new categories, and summarized the categories as integrated themes.

## Results

**Fig. 2:**
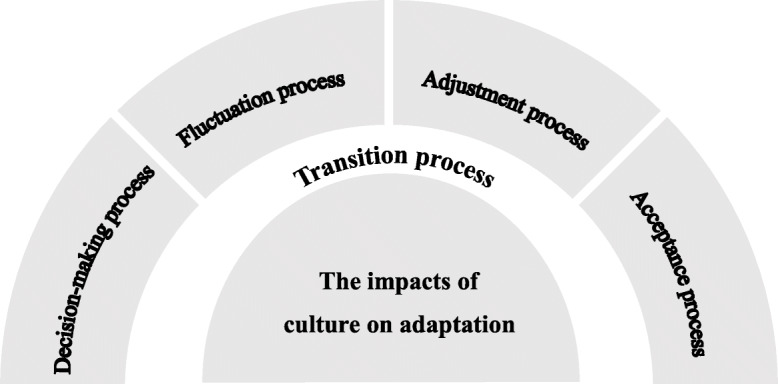
The adaptation of older people’s transition to the RCFs

Categorization is a process of analysing and identifying common themes from the findings of the qualitative studies. These common themes are further categorised to arrive at a synthesised finding. In one study [17], the form of themes was ‘A versus B’ and changed to two themes‘A’ and ‘B’, which facilitated grouping. For example, ‘Ties to the past versus starting a new’ was changed into two findings: ‘Ties to the past’ and ‘starting a new’. Forty-three well-defined findings were extracted, and similar findings were grouped into six new categories. Category1: religion and ‘God’ promoted adaptation; Category2: collectivity and harmony as principles of relationship building; Category3: the decision-making process; Category4: the fluctuation process; Category5: the adjustment process; and Category6: the acceptance process. Then, the six categories were integrated into two synthesized findings: synthesized finding1: the impacts of culture on adaptation, and synthesized finding2: the transition process. (Table 4), (Fig. 2).

**Table 4:** Synthesized findings

Synthesized finding	Category	Finding	Illustration
Synthesized finding1: The impacts of culture on adaptation	Category1 religion and ‘God’ promoting adaptation	Finding1: Renewing	Filipino elderly expressed how a deeper relationship with God commenced as they journeyed through life in the nursing home: “I pray because I know that no one else will help me only God will Being prayerful helps me to adjust [20]
		Finding2: Connecting with a personal philosophy	I am a great believer in God’s plan and implied that they could influence their responses to this new life. They expressed tolerance and acceptance, starting ‘It’s God’s will’ [9]
	Category2: Collectivity and harmony as principles of relationship building	Finding3: Normalizing	Meeting collective needs was also discussed in relation to the Chinese culture of living and eating together as a big family.:If there is a vacancy in the bathroom, you can go ahead to take a bath. If the bathrooms are fully occupied, you have to wait until others had finished. It is the same as living at home—you also have to take turns for bathing. Now, you just live in a bigger room with more neighbors [10].
Synthesized finding 2: The Transition process	Category3: The Decision-making Process	Finding4: Self-motivated move versus familial encouragement	1We talked about moving before I broke my hip. I couldn’t do the work – snow and grass 2My daughter brought me here because I needed supervision. I had fallen at home, and I laid there for two days [17]
		Finding5: Not feeling confident in their decision to move;	Decision to move: I don’t know why I’m here vs. it was a difficult decision [21]
		Finding6: No other options	Oh, I get short of breath. I have to take pills in order to breathe. Sick most of the time. .. so this is the place to be. What can you do when you are alone. I was alone in the small apartment. And back and forth, back and forth to the hospital.. .. You know it was a bad life going back and forth. Now I’m here. And I stay here [22]
		Finding7: Experiencing some difficulties in incorporating this transition into their life stories.	Participants discussed health difficulties restricting their ability to cope alone, often justifying requiring extrafamilial support by highlighting that they would not need to be there if their health were better [21].
	Category4: The Fluctuation Process	Finding8: Ties to the past	The pieces I wanted were brought up here. I knew everything would fit. So my apartment I am happy with. It is, to me, very attractive and has my own favorite things. It meant sorting out and getting rid of a lot and saving some that I did not have the heart to get rid of. But anyway, my apartment is very pretty and that has a lot to do with me being happy here [17].
		Finding9: Independence	They realized that while this transition involved giving up some of their independence such as driving and cooking, [17]
		Finding10: Overwhelmed phase	I get awful lonely and depressed. I wish I could be home. I forget a lot of things, my mind isn’t working the way it should. I go to a room and forget why I went there. I’m afraid if I leave my room. I’ll get lost and won’t find my way back. I don’t want to be a burden to my daughter and her husband [7]
		Finding11: Becoming homeless	Four months I was there, and loved every minute of it, except I was so busy trying to get everything settled. I don’t regret one minute being over there. I want to go back to my apartment; it’s a whole house full of memories. The only thing I’ve regretted is falling and being where I am now [nursing home]. You see the whole story is that if you can’t do for yourself, they have different places for you [18].
		Finding12: Being cut off;	I always say that everything one possesses has its own story. It’s a memento for something. We’re forced to let go of the things we had before [19].
		Finding13: Being restricted	It was only when I arrived here that I realised that I couldn’t cope anymore. I used to like to cook, to invite friends. I could no longer do any of that [19].
		Finding14: Reminiscing phase	Each of the participants felt an urge to retell the story behind his present life in a nursing home.: All I can say I that I’m not satisfied because my family wasn’t here [20].
		Finding15: Emotional reactions	I don’t sleep … I just stare at the wall. A feeling of shock was expressed [9].
		Finding16:Reflecting key plots of ‘control’, ‘power’, ‘identity’ and ‘uncertainty’ interwoven throughout their narratives;	I miss the privacy and freedom of my own home. I used to control my pension [...] they take all that off you to come here you know Uncertainty: Then one day I had a stroke. Apparently () It never occurred to me that I had one [21].
		Finding17: Living in constant fear of losing their memory	Participants’ fears around losing their memory appeared to impact on their behaviour, with individuals describing ‘clinging onto’ their memory [21]
		Finding18: Limited expectations for their future	Well, I’ll grow old here, people do die here, you know, every so often. I think they just take them out in the middle of the night and cremate them, you know [21]
		Finding19: This is who I am,	That is the only reason why I miss my car. If I was bored, I just sat in my car and went up there [to his weekend cottage] for a few days [23]
		Finding20: Disdain	There are a lot of people around here whose minds are not very good; they don’t belong in assisted living anymore. At the dinner table, they don’t mind their manners. One lady uses the tablecloth to wipe her nose [17]
		Finding21: Maladjustment	Although they didn’t miss the upkeep and repairs, it seemed so expensive to make monthly payments. They also found it difficult to adjust to paying for individual services such as medication distribution, assistance with personal hygiene, and bandage changing [17]
		Finding22: Recommencing phase	Instigation of a new phase of life in a nursing home is perceived by the participants as being Stressful: Here, I am not allowed to invite. There are many restrictions. You have to talk to the directors first. In my home I can do everything [20]
	Category5: The Adjustment Process	Result23: Affection	The help here is all very concerned for you and the people, the residents. If anyone hurts, the rest of the people feel it too [17]
		Finding24: Adjustment	It is a matter of attitude. If you decide that it is an adventure, the next stage in life, then you accept it and go on from there [17].
		Finding25: Getting settled and learning the ropes;	I’m acquainted with all these nurses down here. They like me and I like them. Cook’s the same way. She always has a little ice cream for me [18].
		Finding26: Being cared for	They provide the meals and we get to choose what we want … I would give the meals four stars [19].
		Finding27: Dependence	Many participants reflected on becoming increasingly dependent on others [17]
		Finding28: Orienting	They therefore used their own efforts to gain a realistic understanding of the dynamics of every aspect of nursing home life: Ms. Ho [the deputy superintendent] told me not to hang dry my clothes in the corridor. Yet, I saw a lot of others were doing this in the evening. I just informally checked this with Ah Mei. I now know that this rule is really not followed [10]
		Finding29: Rationalizing	They began to soften the blow by downplaying the negative so as to make day-to-day life easier within the limits of nursing home living.: Hey, you can just sit over here for a while and you can hear how some of them [other residents] are talking about missing who and who. We are all like that. It is only abnormal if you don’t miss someone or something [10]
		Finding30: Reinforcing phase	Numerous persons really dole out some goods... Every day, we receive visitors. They give us food so we never run out of food here. Eventually, I became a lot better and happy [20]
		Finding31: Recapturing phase	Respondent’s narrations, when asked about the similarities between their previous home and where they presently live, were often infused with a sense of contentment and ease: Yes, everything’s here, you can’t look for anything else—there are clothes, chairs, beds... you won’t look for anything [20]
		Finding32: Reflecting on their situation	Appraisals of facility and their experience in it ranged from disapproval to ringing endorsement [9].
		Finding33: Downplaying the negative	There was evidence that minimizing negative aspects of their present situations was not only due to a reluctance to complain to an outsider [22]
		Finding34: Adjusting my daily occupations	I found it difficult to live because I didn’t have my own life … but I had to adjust to the extent … that it wasn’t possible [to live her own life]. And now it is, and that’s what matters [23].
		Finding35: Recognizing the ambivalence	I don’t think there is any other place like this that’s any better as far as old people are concerned. It’s not like home. But then I don’t think there is any place that is a better home for old people my age [22].
	Category6: The Acceptance Process	Finding36: Starting a new	Many participants agreed they had made new friends and felt “like a family in their new location. “We watch out for each other, and I try to hold the elevator for those who need more time [17]
		Finding37: Initial accept phase	Nobody can help me but myself. You’ve got to pick up your boots and do it. As long as I can keep my sense of humour and keep walking it should be all right. It’s starting to be more like home [7].
		Finding38: Creating a place	Creating a place was a constitutive pattern: “I’ve met a lot of people here...they treat you right.” This demonstrates the significance of creating a place by developing new memories, new friends, and new neighbors. Two residents who were asked to change their rooms declined and adamantly stated, “This is my place, and I’m not moving. My family knows where I am. I’m staying here [18]
		Finding39: Moving on;	It’s okay at this age and given my heart condition – things weren’t very good before. Yes, it’s okay. I’m in the right place, there’s care if I need it and I’m well looked after [19]
		Finding40: Stabilizing	The central theme thus identified was resignation from previous life: to be simple, peaceful, take things easy, eat, and rest more: It’s fate that brings us here—in this very same nursing home! I have to accept this [10].
		Finding41: Rekindling Phase	Rekindling is demonstrated by the elderly when they restore a sense of being at home in the institution as acceptance and contentment is reached: Yes, it’s hard but after a long time of being here, we were able to adjust, we are able to recover. Whatever possession you have, be contented with it. That’s what I feel [20].
		Finding42: An act of will	So I got in my mind that this is it. I’ve got to be here. I’ve got to live with it. I made out my mind no matter what, it’s all right, it’s good.. .. It depends upon the person [22].
		Finding43: The value of health	Janez felt that the surroundings of his weekend cottage had positive health effects for him.at [the weekend cottage] I have pine trees, I have greenness around me and I can really say that I can see better. That air influences my eyesight [23].

### Synthesized finding 1

The impacts of culture on adaptation contained two items: religion and ‘God’ promoting adaptation, and collectivity and harmony as principles of relationship building.

#### Religion and ‘god’ promoting adaptation

Filipino older adults thought God accompanied and supported them through life in the nursing home. When they prayed, they sensed an easing of their burdens and a sense of guidance. They stated that they experienced a deeper relationship with God when they spent their life in the nursing home [20].

Being prayerful helps me to adjust.

I feel light whenever I pray, while I feel burdened when I do not.

The Canadians in one study thought moving to an RCF was God’s arrangement and tried to accept it, as a religious coping strategy. Their belief influenced their response to the new environment. The Canadians stated that their past, present and intended behaviours were congruent with their belief systems and expressed tolerance and acceptance [9].

It is God’s will

You can not have everything.

Life is what you make it.

I am a great believer in God’s plan.

#### Collectivity and harmony as principles of relationship building

The values of collectivity and harmony serve as principles for relationship building. Many older adults understand this and use these principles in daily life to adapt to new environments. Chinese older adults were eager to learn the rules and regulations of nursing homes to maintain harmony. Therefore, they obeyed these regulations and attempted to fit in by repatterning their lifestyles and daily routines. In terms of communal living, older adults expressed their understanding of meeting collective needs rather than individual needs in dealing with various problems arising from living together. Their past experience living with many families enabled them to easily cope with the current need to share facilities [10].

If there is a vacancy in the bathroom, you can go ahead to take a bath. If the bathrooms are fully occupied, you have to wait until others have finished. It is the same as living at home—you also have to take turns for bathing. Now, you just live in a bigger room with more neighbours.

### Synthesized finding 2

The transition process included four stages: the decision-making process, the fluctuation process, the adjustment process, and the acceptance process.

#### The decision-making process

Residents have many reasons for making the transition to the RCFs. In the studies, some older adults cited their worsening health and the inability to take care of themselves [17]; while others cited their personal safety when they lived alone at home [17, 22]; They also explained that a nursing home was a place for rest [22]. These reasons motivated their move. In addition, the move eased family members’ burden [22]. Unsafe environments, family relationships, and advice from hospital staff led to the ultimate decision to move [21].

We talked about moving before I broke my hip. I couldn’t do the work – snow and grass [17].

Oh, I get short of breath. I have to take pills in order to breathe. Sick most of the time . . . so this is the place to be. What can you do when you are alone. I was alone in the small apartment. In addition, back and forth to the hospital. . . . You know it was a bad [22]

Participants emphasized their control over their relocation decision, evaluated their options, retained the right to return home, and questioned the decision with uncertainty [21]. Some had limited freedom to decide where or when to live and family members directly made decisions [21].

At the back of my mind was, I’m only coming here for six weeks and if it’s all right I’ll stay, if it isn’t, I’ll go back [21].

My son made [the decision] for me [...] he put me in a care home [21].

#### The fluctuation process

Nine studies covered the fluctuation process. Older adults wanted to share the story behind their present life and missed the past [20]. Ties to the family were important and influenced the adjustment [17, 20]. The past made a deep impression on the participants. Unfortunately, some ties to the past could not compensate for the new environment. Therefore, the older adults had feelings of being cut-off, faced loss, discontinuity and a void, and experienced little or no participation in the new life [19].

The pieces I wanted were brought up here. I knew everything would fit. So my apartment I am happy with. It is, to me, very attractive and has my own favourite things. It meant sorting out and getting rid of a lot and saving some that I did not have the heart to get rid of. However, anyway, my apartment is very pretty and that has a lot to do with me being happy here [17]

Most residents experienced an emotional shock during the initial period of relocation. They felt overwhelmed, fearful, homeless, a loss of control, unhappy, resistant, and etc. [7, 9, 17, 21].

I don’t sleep … I just stare at the wall [9].

I get awful lonely and depressed. I wish I could be home. I forget a lot of things, my mind isn’t working the way it should. I go to a room and forget why I went there. I’m afraid if I leave my room. I’ll get lost and won’t find my way back. I don’t want to be a burden to my daughter and her husband [7].

Activities in nursing homes followed standardized procedures, which could constrain the residents [19]. Many complained about losing independence, such as driving and cooking [20, 23]. Some disdained social relationships because they were used to being alone or found it hard to interact with residents with dementia [17].

#### The adjustment process

Residents were cared for by staff members and depended on others. They missed the support and security they had felt at home. Then, they started to reflect on their situation, rationalized their attitudes, tended to downplay the negative, and took strategies to adjust to the new environment [9, 10, 22]. The older adults gradually realized that there were certain aspects of life that could not be managed in the way they would like; they began to downplay the negative aspects of the difficult transition to make daily life easier in RCFs [10]. Their appraisals of the facility and their experiences there ranged from disapproval to ringing endorsement [9]. They came to adjust to the rules and regulations, changed their daily occupations and settled in [10, 18, 23]. The residents attempted to become known and to know others in the unfamiliar surroundings [18]. Gradually, they recognized the benefits of the RCFs and became content with the service [20]. They welcomed more social activities and developed affectionate relationships with other residents [17].

I’m acquainted with all these nurses down here. They like me and I like them. Cook’s the same way. She always has a little ice cream for me [18].

They provide the meals and we get to choose what we want … I would give the meals four stars [19].

I don’t think there is any other place like this that’s any better as far as old people are concerned. It’s not like home. But then I don’t think there is any place that is a better home for old people my age [22].

#### The acceptance process

The acceptance process was the final phase in the transition to RCFs. Older adults made efforts to adjust to the new environment [22]. They felt new hope again because of the improvement in their physical conditions [23]. They expressed the hope of being happy with their situation [22]. Therefore, they were more positive and thought they could move on in life [19]. They started to accept their new life and create more stability, as they had at home [7, 10, 19, 22]. The older adults made new memories, new friends, and new neighbours [7, 18]. They also found new ways to pass the time [20]. They focused on others beyond themselves, took control of the situation, and started to feel as if they were at home [7]. Some talked a lot about ‘making their minds up’ in the context of accepting their situation,

So I got in my mind that this is it. I’ve got to be here. I’ve got to live with it. I made out my mind no matter what, it’s all right, it’s good. . . . It depends upon the person [22].

Yes, it’s hard but after a long time of being here, we were able to adjust, we are able to recover. Whatever possession you have, be contented with it. That’s what I feel [20].

## Discussion

There were two main findings including the impacts of culture on adaptation and the transition process (the decision-making process, the fluctuation process, the adjustment process, and the acceptance process). We identified two significant results involving how specifically culture influenced residents’ adjustment and extended the understanding of the transition process. A previous study reported that cultural considerations were important factors in assessing the quality of a nursing home [25]. The perception of different cultural values was important to both residents and caregivers [9]. To our knowledge, this study also contributed to the literature by observing that the decision-making process was first incorporated into the transition process, which required the participation of older people, family members, and nursing staff together.

The cultural impacts on the adaptation to RCFs were reported in three studies from three countries (Canada, the Philippines, China) [9, 10, 20]. By recognizing the cultural factors influencing the adaptation of residents during relocation, care providers might be better able to meet the previously unconsidered needs for cultural consistency. It is essential to gain knowledge about residents’ cultural values, beliefs and experiences to provide adequate care. Therefore, individualized care plans could be written culturally specific. God was an important figure in the religious and personal belief system of Canadians, helping them make sense of their lives and giving meaning to their present situation, a new phase at the beginning of adaptation [9]. The beliefs they had held throughout their lives enabled them to remain true to themselves, despite their changed circumstances [9]. The Philippines, located in East Asia, is not only the first country in Asia to be influenced by Western culture but also one of the centres of cultural integration between East and West [26]. The main religious beliefs of the Filipinos are influenced by Spain [26]. The admission to a nursing home was stressful to the Filipinos because of their traditional familism; however, they experienced an easing of their burdens and a sense of guidance when they felt God accompanying them [20]. It has been suggested that care providers be attentive to spiritual aspects of residents’ lives and assist them in maintaining an optimistic view of the RCF and life as a whole [20]. ‘Familism’ originated from China [27]. Chinese residents adjusted themselves to relocation using the principles of ‘collectivity and harmony’ [10]. They believed that the group and collectivism are the basis of the strength of a family, and they treated the RCF as a large family [12]. Confucianism emphasizes maintaining harmony in social relationships [28]. Compared with Americans, who emphasize individualism and privacy, Chinese older adults accepted the rules and regulations without difficulties [29].

The process of moving to an RCF requires residents to make complex and important decisions [30]. The decision-making process is the first step of the transition to the RCFs. During this period, the degree of preparation for relocation is very important, for it determines the outcome of the overall transition. The residents whose admission to a nursing home was unplanned had poor adaptation [19]. Some older adults who felt excluded from the process reported a decrease in psychological well-being [30]. The decision-making process mainly consisted of two elements: the reasons for relocation and behaviors of the decision-making for placement. Similar results have been found in the study, which revealed four stages: initiating the placement decision (mainly reasons), assessing and weighing the decision, finalizing the decision, and evaluating the decision [31]. Furthermore, traditional culture influenced some Asian countries, such as the Philippines and China [10, 20]. Most caregivers wish to take care of their family members with chronic conditions at home and regard the RCFs as a last resort. In China, the traditional virtue is a heritage of Confucian philosophy that highlights the significance of filial piety, affecting RCFs admission. Chinese older adults traditionally hoped to be taken care of by their children at home and had to overcome many difficulties in reaching the relocation decisions [31].

Even at the stage when the decision is fully made, for example, regarding the choice of a facility close to home and with a good quality of service, many older people encounter a second stage of fluctuation, a hard period, showing maladjustment. The majority of residents had emotional reactions regardless of their nationality. Older people whose admission was unplanned experienced tougher times and more challenges during the transition than people who had planned their admission beforehand [7]. The reduction in previous communication networks led to separation, a sense of isolation and loneliness [19]. The reasons for difficulties in communication with other residents involved illnesses, visual and hearing impairment, personal preference, etc. Memories associated with home and the past are precious in the minds of the residents. Therefore, familiar pictures, furniture and even pets were allowed to be brought into some RCFs, creating a homelike atmosphere, which helped ease the transition in Western countries [17]. However, RCFs in Taiwan, China were designed to be more like a hospital, because of their constricted space [12].

As there was a decline in function and increasing illness, residents were becoming more dependent on caregivers. After the fluctuation process, they attempted to focus on the present rather than the previous: valuing the benefits of the facilities, exhibiting more of a positive attitude, learning the rules and regulations, and developing individual coping strategies. When asked about the similarities between their previous home and where they presently live, residents were often filled with a sense of contentment and comfort during this period [20]. During the adjustment process, the older people attempted to develop new relationships with other residents, establishing new friendships and gaining mutual companionship, although sometimes they found it hard to communicate with people who were cognitively impaired. However, there were few conversations between the residents and staff members as a result of work demands, which limited opportunities for verbal interaction [18]. Positive relationships between residents and staff are a central part of quality care [32].

The final stage was ‘the acceptance process’, during which residents became more confident, created new meanings, treated the place like home and took more control of their personal lives, which was also an indicator of good adaptation.

Nursing staff members play a pivotal role in educating, advocating, and supporting residents’ transition to RCFs. Nurses should understand that the process of adaptation is dynamic, and tailored interventions should be considered to meet residents’ needs in their own time. There are four key points for promoting adaptation: encouraging residents to express their feelings; establishing trust and conveying respect towards residents; interacting with residents as much as possible; and increasing family involvement.

Nursing staff members should recognize that the experience of moving to an RCF is different for each individual [17]. The nursing home should designate a staff member or volunteer who has good interviewing skills and is empathic and supportive to communicate with older people about their experience at a regularly scheduled time during the early stage of the transition [7]. We could expand the scope of social support and recruit volunteers such as university students and healthily older adults in community. Using story sharing as a focus, programs could be conducted by a transitional care team consisting of the older people, family members, nursing staff, physician, activities director, social worker, etc. [18]. The content of the stories to be shared includes the feelings about the relocation, the experience of transition, meanings of the changes, past and current life stories, and even food likes and dislikes [17, 18, 20, 23]. Story sharing can promote the development of reciprocal relationships [18].

### Implications for practices and limitations

The adjustment of older adults in the transition to RCFs should be assessed in the cultural context. Care providers should pay attention to the spiritual aspects of residents’ lives and cultural copying strategies to facilitate adaptation. Care plans should be initiated in the stage of the decision-making process, not postadmission.

The grey literature was not included in the literature search in this study. The studies included were limited to those published in the English language. There was a limitation for accurate definition for ‘residential care facility’ because these facility terms are not interchangeable as they have different entry criteria, different staffing, and different services offered. Different cultural data and information may not be accurately interpreted, which might cause bias.

## Conclusions

The decision-making process is the first part of the transition process; this finding extends the scope of the transition to RCFs. Thus, tailored interventions such as culturally congruent care should be considered to meet the unique needs of older persons and facilitate their transition to the RCF. Older adults’ belief systems should be fully respected. RCFs can provide prayer rooms and create a home-like atmosphere. Care plans should be made by residents, family members, and staff members together at the beginning of the decision-making process.

## Supplementary Information

**Additional file 1.** Search strategies.

**Additional file 2.** List of study findings.

## Data Availability

All data generated or analysed in this study were included in this published article. The data was presented in Tables 3, 4.

## Abbreviation

RCFs: Residential care facilities
