# Supplementary material for: The adaptation of older adults’ transition to residential care facilities and cultural factors: a meta-synthesis
Source: BMC Geriatr. 2021 Jan 18;21:64. doi: 10.1186/s12877-020-01987-w (PMC7818340; doi:10.1186/s12877-020-01987-w)
Supplement: Supplementary file 1 — Additional file 1. Search strategies. [file 12877_2020_1987_MOESM1_ESM.docx]

**Search strategies**

Pubmed: 43 articles

Cochrane:275 articles

CINAHL :99 articles

Web of Science:36 articles

Embase: 18 articles

PsycInfo:22

Summary: 493 articles

**PUBMED**

1 (((((((relocation[Title/Abstract]) OR transition[Title/Abstract]) OR admission[Title/Abstract])) AND (((("Residential Facilities"[Mesh]) OR "Homes for the Aged"[Mesh]) OR "Long-Term Care"[Mesh]) OR ((((aged care home[Title/Abstract]) OR long-term care facility[Title/Abstract]) OR LTC facilities[Title/Abstract]) OR residential aged care facility[Title/Abstract]))) AND ((("Social Adjustment"[Mesh])) OR ((((((adaptation[Title/Abstract]) OR adjustment[Title/Abstract]) OR psychosocial changes[Title/Abstract]) OR psychosocial adjustment[Title/Abstract]) OR adapt*[Title/Abstract]) OR adjust*[Title/Abstract])))) AND (("Aged"[Mesh]) OR ((((((older adults[Title/Abstract]) OR older persons[Title/Abstract]) OR elderly individuals[Title/Abstract]) OR elderly people[Title/Abstract]) OR elderly residents[Title/Abstract]) OR residents[Title/Abstract]))

2nursing methodology [tiab] OR case study [tiab] OR constant comparison [tiab] OR content analysis[tiab] OR descriptive study [tiab] OR discourse analysis[tiab] OR ethnography [tiab] OR exploratory [tiab] OR feminist [tiab] OR focus group

[tiab] OR grounded theory [tiab] OR hermeneutic [tiab] OR interview[tiab] OR narrative [tiab] OR naturalistic[tiab] OR participant observation [tiab] OR phenomenology [tiab] OR qualitative method [tiab] OR qualitative research [tiab] ORqualitative study [tiab] OR thematic analysis [tiab]

3 #1 AND # 2 43

**Cochrane**

1 Mesh descriptor:[Emotional Adjustment] explode all trees 48

2 Mesh descriptor:[Social Adjustment] explode all trees 911

3ti,ab,kw(adaptation OR adjustment) OR psychosocial changes OR psychosocial adjustment OR adapt* or adjust*) 102889

4Mesh descriptor:[Residential Facilities ] explode all trees 1696

5 Mesh descriptor:[Homes for the Aged] explode all trees 608

6Mesh descriptor:[Long-term Care] explode all trees 1107

7ti,ab,kw( aged care home or long-term care facility or LTC facilities or residential aged care facility) 102702

8Mesh descriptor:[Aged] explode all trees 7469

9 ti,ab,kw:( older adults OR older persons OR elderly people OR elderly residents OR residents) 66997

10 ti,ab,kw (relocation or transition or move or admission)

11 #1 OR #2 OR #3 1234512

12 #4 OR #5 OR #6 OR #7 103296

13 #8 OR #9 73025

14 #11 AND #12 AND #13 AND #10 937

15 “nursing methodology” OR “case study” OR “constant comparison” OR “content analysis” OR “descriptive study” OR “discourse analysis” OR ethnography OR exploratory OR feminist OR “ focus group” OR “grounded theory” OR hermeneutic ) OR AB ( “nursing methodology” OR “case study” OR “constant comparison” OR “content analysis” OR “descriptive study” OR “discourse analysis” OR ethnography OR exploratory OR feminist OR “ focus group” OR “grounded theory” OR hermeneutic 26681

16 #14 AND # 15 275

**Web of Science**

1 TS:("Social Adjustment" OR adaptation OR adjustment OR “psychosocial changes” OR “psychosocial adjustment” OR adapt* OR adjust*) OR TI:(("Social Adjustment" OR adaptation OR adjustment OR “psychosocial changes” OR “psychosocial adjustment” OR adapt* OR adjust*)) 1404988

2 TS: (relocation OR transition OR admission OR move) OR TI: (relocation OR transition OR admission OR move) 1548871

3 TS:("Residential Facilities" OR "Homes for the Aged" OR "Long-Term Care" OR “aged care home” OR “long-term care facility” OR “LTC facilities” OR “residential aged care facility”)

OR TI:("Residential Facilities" OR "Homes for the Aged" OR "Long-Term Care" OR “aged care home” OR “long-term care facility” OR “LTC facilities” OR “residential aged care facility”) 14368

4 TS:("Aged" OR “older adults” OR “older persons” OR “elderly individuals” OR “elderly people” OR “elderly residents” OR residents） OR TI:("Aged" OR “older adults” OR “older persons” OR “elderly individuals” OR “elderly people” OR “elderly residents” OR residents) 609450

5 TS:(“nursing methodology” OR “case study” OR “constant comparison” OR “content analysis” OR “descriptive study” OR “discourse analysis” OR ethnography OR exploratory OR feminist OR “focus group” OR “grounded theory” OR hermeneutic OR interview OR narrative OR naturalistic OR “participant observation” OR phenomenology OR “qualitative method” OR “qualitative research” OR “qualitative study” OR “thematic analysis” ) OR TI:(“nursing methodology” OR “case study” OR “constant comparison” OR “content analysis” OR “descriptive study” OR “discourse analysis” OR ethnography OR exploratory OR feminist OR “focus group” OR “grounded theory” OR hermeneutic OR interview OR narrative OR naturalistic OR “participant observation” OR phenomenology OR “qualitative method” OR “qualitative research” OR “qualitative study” OR “thematic analysis” ) 544794

6 #1 AND #2 AND #3 AND #4 AND #5 36

**CINAHL Complete**

1. (MH "Adaptation, Psychological+") OR "adaptation" OR (MH "Adaptation, Physiological+") 64564

# 2(MH "Social Adjustment") OR "Social Adjustment"  5087

3TI ( “psychosocial changes” OR “psychosocial adjustment ” OR adapt* OR adjust* ) OR AB ( “psychosocial changes” OR “psychosocial adjustment ” OR adapt* OR adjust* ) 297406

4 S1 OR S2 OR S3  224371

5 (MH "Residential Facilities+") OR "Residential Facilities" 34707

6(MH "Long Term Care") OR "Long-Term Care"  75595

7 (MH "Nursing Homes+") OR "Nursing Homes" 34811

8 TI ( "Homes for the Aged" OR "long-term care facility" OR "LTC facilities" OR "residential aged care facility" ) OR AB ( "Homes for the Aged" OR "long-term care facility" OR "LTC facilities" OR "residential aged care facility" ) 1114

9 S5 OR S6 OR S7 OR S8  54364

10 (MH "Relocation") OR "Relocation" OR (MH "Relocation Stress Syndrome (NANDA)")  3069

11 TI ( transition OR admission OR move ) OR AB ( transition OR admission OR move )  146989

12 S10 OR S11  97655

13(MH "Aged+") OR "Aged" ， 10111572
14 TI ( older adults or elderly or seniors or "older persons" or "residents" ) OR AB ( older adults or elderly or seniors or "older persons" or "residents" )  230484

15 S13 OR S14  715780

16 (“nursing methodology” OR “case study” OR “constant comparison” OR “content analysis” OR “descriptive study” OR “discourse analysis” OR ethnography OR exploratory OR feminist OR “focus group” OR “grounded theory” OR hermeneutic OR interview OR narrative OR naturalistic OR “participant observation” OR phenomenology OR “qualitative method” OR “qualitative research” OR “qualitative study” OR “thematic analysis” ) 331609

30 S4 AND S9 AND S12 AND S15 AND S16  99

**EMBASE**

1. 'adaptation'/exp OR 'adaptation' OR 'social adaptation'/exp OR 'social adaptation' 412414
2. 'adjustment'/exp OR 'adjustment' 231673
3. 'psychosocial changes’ 399
4. 'social psychology'.mp. or exp social psychology/ 132851
5. 'adapt*' 786201
6. 'adjust*' 871301
7. #1 OR #2 OR #3 OR #4 OR #5 OR #6 1850987

8 'relocation'/exp OR 'relocation' 7358

9 'transition'/exp OR 'transition' 420336

10 'admission':ab,ti 419037

11 #8 OR #9 OR #10 853240

12 aged/ or 'aged'.mp. 4524147

13 'older adults': 94556

14'older persons:':ti,ab,kw 12413

15'elderly individuals:':ti,ab,kw 10023

16'elderly people':ti,ab,kw 26445

17 'elderly residents':ab,ti 1678

18 'residents':ti,ab,kw 134848

19 #12 OR#OR3#OR14#OR15#OR16#OR#17OR#18 4642147

20'residential facilities':ti,ab,kw: 977

21 'home for the aged'/exp OR 'home for the aged' 12592

1. 'long term care'.mp. or long term care/ 1765257

23 'nursing home':ti,ab,kw 28495

24 ''long term care facility.':ti,ab,kw 2588

1. 'residential home':ti,ab,kw 477

26 'ltc facilities':ti,ab,kw 649

27 'residential aged care facility':ti,ab,kw' 177

28#20 OR #21 OR #22 OR #23 OR #24 OR #25 OR #26 OR #27 1797282

1. 'qualitative research/':ti,ab,kw OR 'qualitative method':ti,ab,kw OR 'qualitative analysis':ti,ab,kw 47587

30 #7 and #11 and #19 and# 28 and# 29 18

**PsycINFO**

# 1TI ("Social Adjustment" OR adaptation OR adjustment OR “psychosocial changes” OR “psychosocial adjustment” OR adapt* OR adjust*) OR AB ("Social Adjustment" OR adaptation OR adjustment OR “psychosocial changes” OR “psychosocial adjustment” OR adapt* OR adjust*) 377,652

# 2 TI(relocation or transition or move or admission) OR AB(relocation or transition or move or admission) 176,743

3 TI ( older adults or elderly or seniors or geriatrics or aged or residents ) OR AB ( older adults or elderly or seniors or geriatrics or aged or residents ) 470,027

4 TI ( older persons or elderly individuals or elderly people or older people ) OR AB ( older persons or elderly individuals or elderly people or older people ) 38,492

5 S2 OR S4  481,853

6 TI ( Residential Care Institutions or Residential Facilities or Long-Term Care or Nursing Homes or aged care home or long term care facility or residential care or LTC facilities or residential aged care facility ) OR AB ( Residential Care Institutions or Residential Facilities or Long-Term Care or Nursing Homes or aged care home or long term care facility or residential care or LTC facilities or residential aged care facility )  31,893

7 TI ( “nursing methodology” OR “case study” OR “constant comparison” OR “content analysis” OR “descriptive study” OR “discourse analysis” OR ethnography OR exploratory OR feminist OR “ focus group” OR “grounded theory” OR hermeneutic ) OR AB ( “nursing methodology” OR “case study” OR “constant comparison” OR “content analysis” OR “descriptive study” OR “discourse analysis” OR ethnography OR exploratory OR feminist OR “ focus group” OR “grounded theory” OR hermeneutic )  252761

8 S1 AND S2 AND S5 AND S6 AND S7  22
