# Supplementary material for: The adaptation of older adults’ transition to residential care facilities and cultural factors: a meta-synthesis
Source: BMC Geriatr. 2021 Jan 18;21:64. doi: 10.1186/s12877-020-01987-w (PMC7818340; doi:10.1186/s12877-020-01987-w)
Supplement: Supplementary file 2 — Additional file 2. List of study findings. [file 12877_2020_1987_MOESM2_ESM.doc]

List of study findings

| Author | Finding | Illustration |
| --- | --- | --- |
| Tracy | Self-motivated move versus familial encouragement | 1We talked about moving before I broke my hip. I couldn’t do the work – snow and grass  2My daughter brought me here because I needed supervision. I had fallen at home, and I laid there for two days (Tracy et al)(p29) |
|  | Ties to the past | The pieces I wanted were brought up here. I knew everything would fit. So my apartment I am happy with. It is, to me, very attractive and has my own favorite things. It meant sorting out and getting rid of a lot and saving some that I did not have the heart to get rid of. But anyway, my apartment is very pretty and that has a lot to do with me being happy here.（Tracy et al）(P30) |
|  | Starting a new | Many participants agreed they had made new friends and felt “like a family in their new location. “We watch out for each other, and I try to hold the elevator for those who need more time(Tracy et al)(P30) |
|  | Independence | They realized that while this transition involved giving up some of their independence such as driving and cooking. (P30) |
|  | Dependence | Many participants reflected on becoming increasingly dependent on others(Tracy et al)(P30) |
|  | Affection | The help here is all very concerned for you and the people, the residents. If anyone hurts, the rest of the people feel it too(Tracy et al)(P30) |
|  | disdain | There are a lot of people around here whose minds are not very good; they don’t belong in assisted living anymore. At the dinner table, they don’t mind their manners. One lady uses the tablecloth to wipe her nose(Tracy et al) |
|  | Adjustment | Although they didn’t miss the upkeep and repairs, it seemed so expensive to make monthly payments. They also found it difficult to adjust to paying for individual services such as medication distribution, assistance with personal hygiene, and bandage changing(Tracy et al) (P30) |
|  | Maladjustment | Although they didn’t miss the upkeep and repairs, it seemed so expensive to make monthly payments. They also found it difficult to adjust to paying for individual services such as medication distribution, assistance with personal hygiene, and bandage changing. (P30) |
| Wilson | Overwhelmed phase | I get awful lonely and depressed. I wish I could be home. I forget a lot of things, my mind isn’t working the way it should. I go to a room and forget why I went there. I’m afraid if I leave my room. I’ll get lost and won’t find my way back. I don’t want to be a burden to my daughter and her husband(Wilson et al) (P866) |
|  | Adjustment | You have to have a positive attitude. It’s all in your disposition, you know. If you are going to be a grouch, be one, but it doesn’t hurt to give someone a smile and talk to them. I chase up and down the hall [in the wheelchair]. They have lots of things for you to do. It’s up to you if you want to do them. If you don’t, you don’t(P868) |
|  | Initial accept phase | Nobody can help me but myself. You’ve got to pick up your boots and do it. As long as I can keep my sense of humour and keep walking it should be all right. It’s starting to be more like home.(Wilson et al).(P869) |
| Heliker | Becoming homeless | Four months I was there, and loved every minute of it, except I was so busy trying to get everything settled. I don’t regret one minute being over there. I want to go back to my apartment; it’s a whole house full of memories. The only thing I’ve regretted is falling and being where I am now [nursing home]. You see the whole story is that if you can’t do for yourself, they have different places for you.(Heliker et al) (P37) |
|  | Getting settled and learning the ropes | I’m acquainted with all these nurses down here. They like me and I like them. Cook’s the same way. She always has a little ice cream for me.(Heliker et al).(P39) |
|  | Creating a place | Creating a place was a constitutive pattern: “I’ve met a lot of people here...they treat you right.” This demonstrates the significance of creating a place by developing new memories, new friends, and new neighbors. Two residents who were asked to change their rooms declined and adamantly stated, “This is my place, and I’m not moving. My family knows where I am. I’m staying here(Heliker et al)(P39) |
| Koppitz | Being cared for; | They provide the meals and we get to choose what we want… I would give the meals four stars.(Koppitz et al).(P6) |
|  | Moving on; | It’s okay at this age and given my heart condition – things weren’t very good before. Yes, it’s okay. I’m in the right place, there’s care if I need it and I’m well looked after(Koppitz et al)(P7) |
|  | Being cut off; | I always say that everything one possesses has its own story. It’s a memento for something. We’re forced to let go of the things we had before.(Koppitz et al)(P4) |
|  | Being restricted | It was only when I arrived here that I realised that I couldn’t cope anymore. I used to like to cook, to invite friends. I could no longer do any of that.(Koppitz et al)(P6) |
| Lee 2002 | Orienting | They therefore used their own efforts to gain a realistic understanding of the dynamics of every aspect of nursing home life: Ms. Ho [the deputy superintendent] told me not to hang dry my clothes in the corridor. Yet, I saw a lot of others were doing this in the evening. I just informally checked this with Ah Mei. I now know that this rule is really not followed(Lee2002 et al)(P671) |
|  | Normalizing | Meeting collective needs was also discussed in relation to the Chinese culture of living and eating together as a big family.:If there is a vacancy in the bathroom, you can go ahead to take a bath. If the bathrooms are fully occupied, you have to wait until others had finished. It is the same as living at home—you also have to take turns for bathing. Now, you just live in a bigger room with more neighbors .(Lee2002 et al).(P671) |
|  | Rationalizing | They began to soften the blow by downplaying the negative so as to make day-to-day life easier within the limits of nursing home living.: Hey, you can just sit over here for a while and you can hear how some of them [other residents] are talking about missing who and who. We are all like that. It is only abnormal if you don’t miss someone or something(Lee 2002et al) (P672) |
|  | Stabilizing | The central theme thus identified was resignation from previous life: to be simple, peaceful, take things easy, eat, and rest more: It’s fate that brings us here—in this very same nursing home! I have to accept this.(Lee 2002et al) . (P672) |
| Guevara | Reminiscing phase | Each of the participants felt an urge to retell the story behind his present life in a nursing home.: All I can say I that I’m not satisfied because my family wasn’t here.  (Guevara et al) |
|  | Recommencing phase | Instigation of a new phase of life in a nursing home is perceived by the participants as being Stressful: Here, I am not allowed to invite. There are many restrictions. You have to talk to the directors first.In my home I can do everything(Guevara et al).(P804) |
|  | Reinforcing phase | Numerous persons really dole out some goods... Every day, we receive visitors. They give us food so we never run out of food here. Eventually, I became a lot better and happy(Guevara et al)(P805) |
|  | Recapturing phase | Respondent’s narrations, when asked about the similarities between their previous home and where they presently live, were often infused with a sense of contentment and ease: Yes, everything’s here, you can’t look for anything else—there are clothes, chairs, beds... you won’t look for anything(Guevara et al)(P805) |
|  | Renewing | Filipino elderly expressed how a deeper relationship with God commenced as they journeyed through life in the nursing home: ‘‘I pray because I know that no one else will help me only God will  Being prayerful helps me to adjust （Guevara et al） (P806) |
|  | Rekindling Phase | Rekindling is demonstrated by the elderly when they restore a sense of being at home in the institution as acceptance and contentment is reached: Yes, it’s hard but after a long time of being here, we were able to adjust, we are able to recover.Whatever possession you have, be contented with it. That’s what I feel.（Guevara et al)(P806) |
| Iwasiw | Emotional reactions | I don’t sleep…I just stare at the wall. A feeling of shock was expressed.(Iwasiw et al)(P384) |
|  | Transition activities | Decision making about admission; Moving out and moving in Trying to make the LTCF feel like home; Maintaining previous relationships and beginning new ones, Fitting in |
|  | Reflecting on their situation | Appraisals of facility and their experience in it ranged from disapproval to ringing endorsement.(Iwasiw et al) (P386) |
|  | Connecting with a personal philosophy | I am a great believer in God’s plan and implied that they could influence their responses to this new life.  They expressed tolerance and acceptance, starting ‘It’s God’s will’(Iwasiw et al) (P386) |
| Lee 2013 | Reflecting key plots of ‘control’, ‘power’, ‘identity’ and ‘uncertainty’ interwoven throughout their narratives | I miss the privacy and freedom of my own home.  I used to control my pension [...] they take all that off you to come here you know  Uncertainty: Then one day I had a stroke. Apparently (­) It never occurred to me that I had one (Lee2013 et al)(P51) |
|  | Experiencing some difficulties in incorporating this transition into their life stories | Participants discussed health difficulties restricting their ability to cope alone, often justifying requiring extrafamilial support by highlighting that they would not need to be there if their health were better. (P51) |
|  | Not feeling confident in their decision to move | Decision to move: I don’t know why I’m here vs. it was a difficult decision(Lee2013et al)(P51) |
|  | Living in constant fear of losing their memory | Participants’ fears around losing their memory appeared to impact on their behaviour, with individuals describing ‘clinging onto’ their memory(Lee2013 et al)(P53) |
|  | Limited expectations for their future | Well, I’ll grow old here, people do die here, you know, every so often. I think they just take them out in the middle of the night and cremate them, you know(Lee2013 et al)(P53) |
| Kahn | Recognizing the ambivalence | I don’t think there is any other place like this that’s any better as far as old people are concerned. It’s not like home. But then I don’t think there is any place that is a better home for old people my age.（Kahn et al）(P122) |
|  | Downplaying the negative | There was evidence that minimizing negative aspects of their present situations was not only due to a reluctance to complain to an outsider（Kahn et al）.(P123) |
|  | No other Options | Oh, I get short of breath. I have to take pills in order to breathe. Sick most of the time . . . so this is the place to be. What can you do when you are alone. I was alone in the small apartment. And back and forth, back and forth to the hospital. . . . You know it was a bad life going back and forth. Now I’m here. And I stay here(P124) |
|  | An act of will | So I got in my mind that this is it. I’ve got to be here. I’ve got to live with it. I made out my mind no matter what, it’s all right, it’s good. . . . It depends upon the person.(Kahn et al)(p128) |
| Križaj | This is who I am | That is the only reason why I miss my car. If I was bored, I just sat in my car and went up there [to his weekend cottage] for a few days(Križaj et al)(P515) |
|  | Adjusting my daily occupations, | I found it difficult to live because I didn’t have my own life…but I  had to adjust to the extent…that it wasn’t possible [to live her own life]. And now it is, and that’s what matters.(Križaj et al) (P516) |
|  | The value of health | Janez felt that the surroundings of his weekend cottage had positive health effects for him.at [the weekend cottage] I have pine trees, I have greenness around me and I can really say that I can see better. That air influences my eyesight.(Križaj et al) (P517) |
